# Advances in invasive micropapillary carcinoma of the breast research: A review

**DOI:** 10.1097/MD.0000000000036631

**Published:** 2024-01-05

**Authors:** Li-hao Cheng, Xiao-jie Yu, Hao Zhang, Hao-Jie Zhang, Zhongming Jia, Xiao-hong Wang

**Affiliations:** a Department of Breast Surgery, Binzhou Medical University Hospital, Binzhou, Shandong, PR China; b Department of Thyroid Surgery, Binzhou Medical University Hospital, Binzhou, Shandong, PR China.

**Keywords:** breast, invasive micropapillary carcinoma, lymph node metastases

## Abstract

Invasive micropapillary carcinoma (IMPC) of the breast represents a rare subtype of breast cancer, accounting for 1% to 2% of all breast cancers worldwide. Although clinically asymptomatic, they are usually detected during routine breast screenings. The common symptoms include breast lumps, skin or nipple changes, and nipple discharge. Histopathologically, IMPCs are characterized by tumor cells forming small papillary-like structures inside the glandular spaces, and arranged in an inverted pattern, with their apex pointing toward the center of the gland. This unique morphological feature is critical for diagnosing these cases. Another notable characteristic is its high propensity for lymph node metastasis (LNM). While the precise mechanism of metastasis is not clear, unique cellular arrangement and cellular interactions with the surrounding environment might promote tumorigenesis and higher node positivity. Hence, proper lymph node dissection and assessment are particularly crucial for this type of breast cancer. This review aims to discuss the recent progress in managing IMPC cases.

## 1. Introduction

Invasive Micropapillary Carcinoma (IMPC) of the breast represents a rare subtype of invasive breast carcinoma, first introduced by Fisher et al in 1980^[1]^ and was renamed IMPC in 1993. Morphologically, the cancerous cells are distributed along micropapillae or mulberry-like structures that lack a fibrovascular core. Clinically, IMPC shows notable malignant characteristics like a significant lymphovascular invasion (LVI) and regional lymph node metastasis (LNM).^[2]^ In 2003, the World Health Organization characterized IMPC as a unique pathological subtype of breast tumors.^[3]^ Thus, IMPC exhibits a heightened level of malignancy when compared with conventional invasive breast carcinomas. This is duly characterized by increased LNM and LVI rates which imply a poorer prognosis. Several biomarkers, such as KL-67, MUC1, and EMA are associated with the pathological progression and prognosis of IMPC. Particularly, MUC1, whose aberrant expression is linked to tumor proliferation and LNM. Other receptor molecules like sLex and CXCR4 are implicated in tumor immune evasion mechanisms. Additionally, a few proteins like plakoglobin, LEF1, and ARID1A help in IMPC progression. Multiple working mechanisms and signaling pathways influencing IMPC progression are cell cycle regulation, metabolic abnormalities, and protein kinase signaling routes. This review aimed to evaluate cutting-edge research developments concerning IMPC and shed light on its intricate biological as well as therapeutic avenues.

### 1.1. Clinical characteristics

IMPC accounts for approximately 2% to 7% of all invasive breast cancers. Teren et al^[4]^ in a study on 6684 invasive breast cancer cases, identified 147 IMPC cases, making up 2.2% of the total cohort. When compared with the more prevalent Invasive Ductal Carcinoma (IDC), its distinctive features include older age at diagnosis, large tumor size, compromised lymph node status as well as increased LVI, estrogen receptor (ER), and Human Epidermal growth factor Receptor 2 (HER-2) positivity rates.^[5]^ A 2019 study by Lewis^[6]^ on 2660 pure IMPC patients revealed that approximately 55.2% of patients showed regional LNM at the time of diagnosis. This observation was supported by Chen^[7]^ and Fu,^[4]^ who reported that IMPC patients often display increased tumor dimensions and LNM rates. Another study by Wang^[8]^ indicated that IMPC patients were younger, exhibited tumors more commonly in the central and upper quadrants, and had higher LNM rates than IDC patients. Hence, a significant proportion of IMPC cases are classified as Grade III in comparison to IDC when examined histopathologically. Thus, the specific proportion of IMPC composition in diagnosis remains a topic of debate. For instance, Zekioglu et al^[9]^ advocated that an IMPC diagnosis should be given when the IMPC component makes up > 75% of the tumor. Conversely, De La Cruz^[10]^ suggested that a threshold of > 33% should be considered while giving the diagnosis of IMPC. Notably, fewer IMPC components within a tumor can become a significant predictor of LNM. Both Wang^[8]^ and Cemal^[11]^ emphasized that the mere presence of IMPC components, irrespective of their size or proportion, was significantly correlated with increased lymphatic infiltration rate, LNM, recurrence, and distant metastasis rates. Another study by Wang^[12]^ compared the clinicopathological features of pure and mixed IMPC and noted that pure IMPC displays more aggressive characteristics and a higher Luminal B subtype ratio, while mixed IMPC was predominantly of the Luminal A subtype. The studies by Paterakos^[13]^ and Nassar^[14]^ highlighted that pure IMPC is associated with enhanced lymph node metastases than mixed IMPC, suggesting that the IMPC components’ influence on LNM might be related to their representation within the tumor. Conversely, Chen et al^[15]^ noted that tumors with < 25% IMPC components still show high LVI and LNM rates. In conclusion, the majority of studies emphasize that IMPC is characterized by its notably aggressive behavior, with increased LNM, recurrence, and distant metastasis risks. Nonetheless, a consensus is still required regarding the exact proportion of IMPC components necessary for a breast cancer diagnosis.

### 1.2. Histopathological characteristics

IMPC represents a distinct morphological subtype of breast cancer. Histologically, it is characterized by small tumor cell clusters surrounding lacunar or spheroidal spaces. The tumor cytoplasm exhibits a granular or eosinophilic nature. A notable feature is the reversed polarity, described as an “inside-out” or “lateral-out” growth pattern. In this configuration, the cell apical surface faces the stroma instead of the lumen. This phenomenon has been supported by the peripheral positivity of EMA (MUC1) immunostaining.^[16]^

Acs^[17]^ reported that the peripheral MUC1/EMA expression in IMPC tumor cell clusters might be associated with tumor progression and lymphatic metastasis. Fu^[18]^ further emphasized that the polarity reversal within these clusters increases their innate potential for invasion and metastasis. Hence, the tumor tends to exhibit an enhanced propensity for lymphatic spread and metastasis. Due to the reversed polarity and the unstructured spaces, there is a substantial lack of cohesion between the tumor cell clusters. Subsequently, this facilitates their separation into smaller clusters or even individual cells and enhances their invasive and metastatic capabilities.

Reversed polarity in IMPC tumor cells demonstrates more aggressive biological properties like pronounced lymphatic infiltration and nodal metastasis.^[19]^ Moreover, the loss of epithelial polarity and cell dedifferentiation in IMPC plays a pivotal role in migration and invasion, thereby enhancing tumor progression.^[20]^

Hashmi^[21]^ comparatively studied IMPC and Invasive Ductal Carcinoma-Not Otherwise Specified (IDC-NOS) and revealed that certain biomarkers had significantly higher positivity rates in IMPC than IDC-NOS, i.e., ER and PR in IMPC showed 86.7% and 73.3% positivity as compared to 60% and 46% in IDC-NOS. The nodal metastasis rate for IMPC was 66.7%, significantly different from IDC-NOS. Additionally, the LVI frequencies in IMPC and IDC-NOS were 77.8% and 24.8%, respectively. Although IMPC accounts for < 1% of all breast cancers, its high positivity rates for PR and ER, along with a predisposition for nodal metastasis and LVI, indicate that while such patients might display a favorable prognosis, IMPC demonstrates more invasive characteristics.

### 1.3. Lymphatic metastasis

#### 1.3.1. Molecular markers of LNM in IMPC.

**Figure 1.**
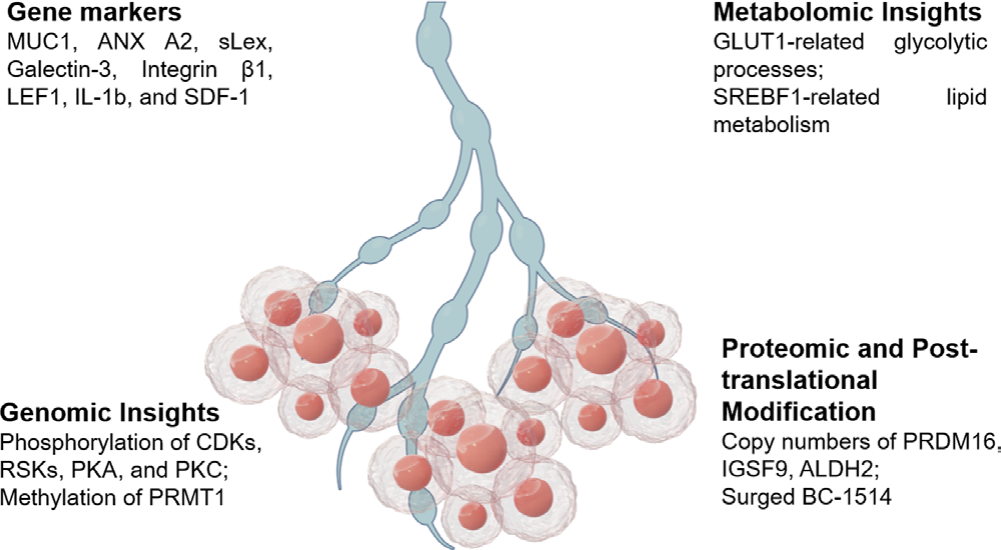
Lymph node metastasis mechanisms include molecular markers, genomic copy number variations, metabolomics, Proteomic and post-translational modification insights.

IMPC has become a key research area due to its propensity for LNM. Thus, several mechanisms underpinning this phenomenon have been elucidated, with biomarkers playing a central role in the identification and understanding of IMPC (Table 1 and Fig. 1). A few prominent markers for IMPC are mucin-1 glycoprotein (MUC1) and epithelial membrane antigen (EMA), while KL-6(Krebs Von den Lungen-6)has also been reported in breast cancer cases.^[30]^ Since Annexin A2 (ANX A2) is recognized as a cellular polarity protein involved in lumen formation, its disrupted functionality results in impaired anti-apoptotic effects and increased apoptosis in tumor cells displaying ANX A2 membrane accumulation.^[22]^ Subsequently, such events trigger polarity disruption which generates detached tumor cells. Thus, the importance of immunohistochemical detection of ANX A2, EMA, and MUC1 as the gold standard markers in IMPC cannot be ruled out.

**Table 1:** The main markers related to lymphatic metastasis in IMPC.

Marker	Significance	Reference
MUC1	Associated with tumor progression and lymphatic metastasis	^[17]^
ANX A2	Involved in impaired anti-apoptotic effects	^[22]^
sLex	masking tumor antigens and interacting with E-selectin, evading immune surveillance	^[23][24]^
Galectin-3	Influencing tumor progression and cell surface polarity	^[25]^
Integrin β1	Involved in Rac1 expression and the reversal of cell polarity	^[26]^
LEF1	Wnt pathway activation	^[27]^
IL-1b	Linked to increased microvessel density	^[28]^
SDF-1	Interacting with CXCR4 on lymphatic endothelial cells	^[29]^

A hallmark of IMPC is cellular polarity reversal and MUC1 expression on the cell basal surface. MUC1 induces tumor cell detachment from the matrix, thereby amplifying its dispersion and causing early LNM.^[23]^ Furthermore, the adhesion molecule sLex is associated with the MUC1/EMA-mediated cellular polarity reversal that leads to enhanced LNM of IMPC.^[24]^

The interaction between sLex, overexpressed on IMPC cell clusters, and E-selectin, highly expressed on lymph endothelial cell surfaces enables lymphatic invasion and LNM in IMPC. Such interactions help tumor cells evade immune surveillance, primarily through NK cell(natural killer cell)interactions. Furthermore, the sialic acid components of Sialyl-Lewis X(sLex) can mask tumor antigens, thereby diminishing the tumor sensitivity to NK cells.^[24]^

A study has indicated that Galectin-3,^[25]^ a protein expressed in both tumor cells and Cancer-Associated Fibroblasts (CAFs), might exert a substantial influence on tumor progression and cell surface polarity. When compared with IDC, IMPC displays significantly elevated Galectin-3 expression levels. This heightened expression in tumor cells correlates with various clinical parameters like microvessel morphology alterations, increased invasive clinical indicators, larger tumors, advanced T stage, and multiple metastatic lymph nodes.

Beyond cellular mechanisms, a multitude of molecular players are also involved in enabling LNM in IMPC. Marchio et al^[31]^ highlighted that an elevated Cyclin D1 expression, pronounced cell proliferation rates, and MYC gene amplification are closely associated with IMPC. A study by Liu^[26]^revealed that in comparison to IDC-NOS, Integrin β1 and Rac1 expressions significantly increase in IMPC; however, Integrin β1 overexpression induces the upregulation of Rac1. Thus, both these proteins are crucial for LNM in IMPC. Furthermore, the positive regulation of β1 Integrin in IMPC is significantly correlated with Rac1 expression and polarity reversal, thereby impacting the prognosis of LNM and disease-free survival. Cui^[28]^ suggested that Interleukin-1 beta (IL-1b) enhances microvessel density in IMPC tumors and LNM progression. Additionally, Cyclooxygenase-2 (COX-2), excessively expressed in various human tumors, exhibits associations with disease progression and multiple adverse prognostic parameters like histological type, tumor recurrence, and regional LNM. Thus, COX-2 plays a crucial role in cancer development through various cellular mechanisms like proliferation, mutation, angiogenesis, immune regulation, and tumor invasion.^[32]^

SDF Q26-1-positive lymphatic vessels are primarily concentrated at the tumor invasive edge that facilitates metastasis. In IMPC tumor cells, the membrane-bound C-X-C chemokine receptor type 4(CXCR4) and stromal cell-derived factor-1(SDF-1)on lymphatic endothelial cell interaction is a pivotal step in lymphatic metastasis.^[29]^ Furthermore, the loss of AT-Rich Interaction Domain 1A(ARID1A) expression is significantly associated with ten-year Overall Survival (OS) and disease-free survival, particularly in the Luminal B subgroup.^[33]^ Lan et al^[34]^ conducted in-depth research on plakoglobin role in IMPC, which is notably overexpressed when compared to IDC-NSTs. Knocking down plakoglobin through shRNA techniques led to the dispersion of IMPC cell clusters and an increased tendency for tumor metastasis. Additionally, the heterogeneous expression of adhesion molecules within primary lesions might promote endogenous tumor cell cluster initiation and tumor metastasis. This phenomenon is also associated with increased PI3K-p85, pAkt, and Bcl-2 expressions in IMPC cell clusters. However, shRNA transfection results show decreased plakoglobin concentration and the PI3K/Akt/BCL-2 pathway activity and suggest that plakoglobin may prevent apoptosis by activating the anti-metastatic pathway. Furthermore, Darin^[27]^ immunohistochemically assessed the β-catenin and LEF1 expressions, prominent Wnt pathway activation markers in IMPC, and demonstrated a significant association between LEF1 expression and LNM, with enhanced LEF1 and β-catenin expressions seen in metastatic lymph nodes as compared to primary tumors. Additionally, a study by Wang^[35]^ found an association between increased LEF1 expression and LNM, distant metastasis, advanced TNM staging, and poorer prognosis in colorectal cancer. Thus, the nuclear expressions of LEF1 and β-catenin increased at the invasive front of colorectal cancer, thereby showcasing their correlation with the upregulation of Wnt target genes, cell mobility, and tumor invasion.

### 1.4. Genomic insights into cancer progression: emphasizing copy number variations (CNVs) and immune evasion in IMPC

Cancer progression, pertaining to genomic copy number variations (CNVs), involves several pivotal molecules and working mechanisms. Few genetic aberrations like single nucleotide variations (SNVs) and CNVs, are intrinsically linked to various tumorigenesis processes, including proliferation, invasion, metastasis, and immune evasion (Table 2 and Fig. 1).

**Table 2:** The genomic insights, and metabolomics insights, proteomic and post-translational modification insights in IMPC.

Marker	Significance	Reference
PRDM16	Reduced copy numbers of IGSF9 and PRDM16 and the increased copy number of ALDH2 were intricately intertwined with lymph node metastasis and reduced survival rates in IMPC patients.	^[36]^
IGSF9		
ALDH2		
BC-1514	Involved in evading immune surveillance	^[37]^
GLUT1	Bolstering glucose uptake and intensifying glycolytic processes.	^[38]^
SREBF1	Involved in heightened activity of lipid metabolism	^[39]^
CDKs	Promoted phosphorylation	^[40]^
RSKs	Promoted phosphorylation modulating the mTORC1/S6K2 signaling axis	^[40]^
PKA	Reduced phosphorylation	^[40]^
PKC	Reduced phosphorylation	^[40]^
PRMT1	Methylation of PRMT1 orchestrating the translation of cell cycle proteins and TNF-associated genes	^[41]^

Jamal^[42]^ discovered that patients with pronounced CNV abnormalities encountered challenging therapeutic trajectories and faced poorer prognoses. Similarly, Shi et al^[36]^ elucidated that LNM was significantly correlated with CNVs than SNVs in IMPC. This underscored the significance of studying CNVs within the genome to decode the invasive and metastatic mechanisms in IMPC cells. They also pinpointed clonal metastatic ancestor cells in primary IMPC, which displayed significant copy number depletion in PR domain-containing 16(PRDM16) and immunoglobulin superfamily member (IGSF9), as well as increased (Acetaldehyde Dehydrogenase 2)ALDH2 copy numbers. Immunohistochemical analysis corroborated that diminished IGSF9 and PRDM16 expressions and the overexpression of ALDH2 were intricately intertwined with LNM and reduced survival rates in IMPC patients. Notably, PRDM16 is a key tumor suppressor gene, with its overexpression efficaciously curtailing MUC4 – a gene overexpressed in IMPC.^[43]^

In an insightful study by Naoki,^[37]^ BC-1514 (C21orf118) emerged as the sole gene in the IMPC region exhibiting an expression surge and increasing 3-fold compared to the ICNST domain. In contrast, a few genes like SAMD13, CAMK2N1, TCF4, TXNIP, RPL5, PJA2, and CD1d displayed diminished expressions in IMPC, thereby signifying their pivotal role in immune evasion. For instance, CD1d interactions with the T-cell receptor on NKT(Natural killer T cell) cell surfaces allow lipid antigens to stimulate anti-tumor responses of NKT cells. Consequently, the declining CD1d expression in IMPC could help the tumor cells in eluding assaults from NKT cells. Moreover, the PJA2 expression might help in the genesis of M1 macrophages, cells that amplify anti-tumor immune responses.

In summation, an intricate tapestry of cellular molecules and mechanisms potentially enables IMPC to surpass the immune system vigilance. Thus, these findings might help in seeking novel research avenues and prospective therapeutic targets for oncological interventions.

### 1.5. Metabolomic perspectives on the pathological mechanisms of IMPC

In the intricate landscape of IMPC, metabolomics has become a pivotal player in understanding its pathological mechanisms (Table 2 and Fig. 1). By using spatial transcriptomics sequencing techniques, Lv^[44]^ elucidated the pronounced metabolic anomalies prevailing within IMPC. Notably, among all stratified IMPC clusters, lipid metabolism exhibited increased activity while the SREBF1 gene was overexpressed. Immunohistochemical analyses confirmed that elevated SREBF1 expression is significantly correlated with LNM and reduced survival rates in IMPC patients.

Intriguingly, FASN, a downstream target gene of SREBF1, displayed a notably higher expression in IMPC when compared with IDC-NOS. Thus, SREBF1 and FASN were significantly correlated with LNM in IMPC. Additionally, SREBF1 functions as a transcription factor after being modulated by the PI3K-mTORC1-AKT signaling pathway.^[39]^ Specifically, mTORC1 enhances the nuclear translocation of SREBF1 through phospholipid-1 phosphorylation. Hence, FASN, acting as the central enzyme in long-chain fatty acid synthesis, takes precedence in maintaining cellular activity.

The dynamic adaptations of tumor cells in modulating metabolic pathways provide them an evolutionary advantage that allows them to proliferate and metastasize even under challenging extracellular conditions. For instance, the fatty acid β-oxidation pathway provides vital energy to tumor cells under energy stress conditions. Such metabolic shifts might be a linchpin in tumor evolution, by helping with their distinct growth patterns and enhanced invasiveness.

Kanae^[38]^ reported that MPCs can upregulate several nutrient transport proteins like ASCT1, ASCT2, LAT1 (L-type amino acid transporter 1), SNAT1, GLUT1, and GLUT2 to address hypoxic and nutrient-deprived microenvironments. Among these, the augmented GLUT1 expression bolsters glucose uptake and intensifies glycolytic processes to meet energy demands. However, the expression of these transport proteins is considerably more pronounced and might elucidate the aggressive nature inherent to IMPC in comparison to non-MPC tumors.

### 1.6. Proteomic and post-translational modification insights in IMPC

While studying the pathological biology of IMPC, proteomics has illuminated novel avenues of understanding, complementing traditional explorations into genes, transcription, and metabolism (Table 2 and Fig. 1). Chen,^[40]^ by employing the sophisticated Liquid Chromatography-Tandem Mass Spectrometry technique, conducted an in-depth proteomic phosphoproteomic analysis of IMPC. Subsequently, a heightened activation state of Cyclin-dependent kinases (CDKs) and the p90 ribosomal S6 kinases (RSKs) was observed within IMPC. However, few CDK family members, notably CDK2, CDK7, and CDK9, were particularly prominent; thus, suggesting potential dysregulation in the cell cycle control mechanisms regarding IMPC. In contrast, kinases associated with cell migration and dynamics, such as Protein Kinase A (PKA) and Protein Kinase C (PKC) families, were significantly suppressed in IMPC. Hence, a few PKC family members like PKCA and PKCD, might exert a tumor-suppressive role in IMPC oncobiology.

While studying the signaling pathways, both TOR and RPS6KB2 exhibited heightened activation. Thus, the phosphorylative inhibition of TSC1/2 by the p90 ribosomal S6 kinase family might modulate the mTORC1/S6K2 signaling axis. Additionally, RHEB overexpression and CAMKK2 hyper-phosphorylation at the S495 residue could initiate the activation of the mTORC1/S6K2 pathway.

Post-translational modifications (PTMs) of proteins play an indispensable role in IMPC pathological mechanics. Specifically, a study^[41]^ on arginine methylation modifications and the metabolic processes of 4-hydroxy-phenylpyruvate within IMPC suggested the role of protein arginine-N-methyltransferase(PRMT1), which induced the translation of cell cycle protein and TNF-associated genes, post-methylation of H4R3 me2a. Given PRMT1 central role in IMPC, it could be a potential therapeutic target for future IMPC treatments.

## 2. Conclusion

IMPC of the breast represents a rare invasive breast carcinoma subtype that shows pronounced malignant features like marked LVI and regional LNM. However, several biological markers are significantly correlated with the malignancy grade of IMPC and prognostic outcomes. From a mechanistic perspective, cell cycle dysregulation, metabolic anomalies, and perturbations in protein kinase signaling cascades might primarily contribute to LNM. From a genetic viewpoint, IMPC progression is due to several genetic mutations, notably SNVs and CNVs. Immunologically, IMPC cells might orchestrate immune evasion after downregulating various specific gene expressions whereas these cells might manifest augmented lipid metabolic activity when seen through a metabolic perspective. Thus, this metabolic adaptability provides survival and metastatic advantages to IMPC, particularly under adverse conditions like energy stress. To conclude, IMPC evolution is intricately linked to myriad molecular and cellular mechanisms. Thus, these insights offer potential targets and future therapeutic intervention approaches for managing breast cancer cases.

## Author contributions

**Conceptualization:** Hao-Jie Zhang, Hao Zhang.

**Visualization:** Zhongming Jia.

**Writing – original draft:** Lihao Cheng, Xiaojie Yu, Hao-Jie Zhang, Hao Zhang.

**Writing – review & editing:** Xiaohong Wang.
